# Absolute Differential Cross-Sections for Elastic Electron Scattering from Sevoflurane Molecule in the Energy Range from 50–300 eV

**DOI:** 10.3390/ijms23010021

**Published:** 2021-12-21

**Authors:** Jelena Vukalović, Jelena B. Maljković, Francisco Blanco, Gustavo García, Branko Predojević, Bratislav P. Marinković

**Affiliations:** 1 Institute of Physics Belgrade, University of Belgrade, Pregrevica 118, 11080 Belgrade, Serbia; jelena.vukovic@pmf.unibl.org (J.V.); jelenam@ipb.ac.rs (J.B.M.); 2 Faculty of Science, University of Banja Luka, Mladena Stojanovića 2, 78000 Banja Luka, Republic of Srpska, Bosnia and Herzegovina; bpredojevic@teol.net; 3 Departamento de Estructura de la Materia Física Térmica y Electrónica e IPARCOS, Universidad Complutense de Madrid, Plaza de Ciencias 1, 28040 Madrid, Spain; pacobr@fis.ucm.es; 4 Instituto de Física Fundamental, Consejo Superior de Investigaciones Científicas, Serrano 113-bis, 28006 Madrid, Spain; g.garcia@csic.es; 5 Centre for Medical Radiation Physics, University of Wollongong, Wollongong, NSW 2522, Australia

**Keywords:** sevoflurane, cross-section, elastic scattering, electrons

## Abstract

We report the results of the measurements and calculations of the absolute differential elastic electron scattering cross-sections (DCSs) from sevoflurane molecule (C_4_H_3_F_7_O). The experimental absolute DCSs for elastic electron scattering were obtained for the incident electron energies from 50 eV to 300 eV, and for scattering angles from 25° to 125° using a crossed electron/target beams setup and the relative flow technique for calibration to the absolute scale. For the calculations, we have used the IAM-SCAR+I method (independent atom model (IAM) applying the screened additivity rule (SCAR) with interference terms included (I)). The molecular cross-sections were obtained from the atomic data by using the SCAR procedure, incorporating interference term corrections, by summing all the relevant atomic amplitudes, including the phase coefficients. In this approach, we obtain the molecular differential scattering cross-section (DCS), which, integrated over the scattered electron angular range, gives the integral scattering cross-section (ICS). Calculated cross-sections agree very well with experimental results, in the whole energy and angular range.

## 1. Introduction

**Figure 1:**
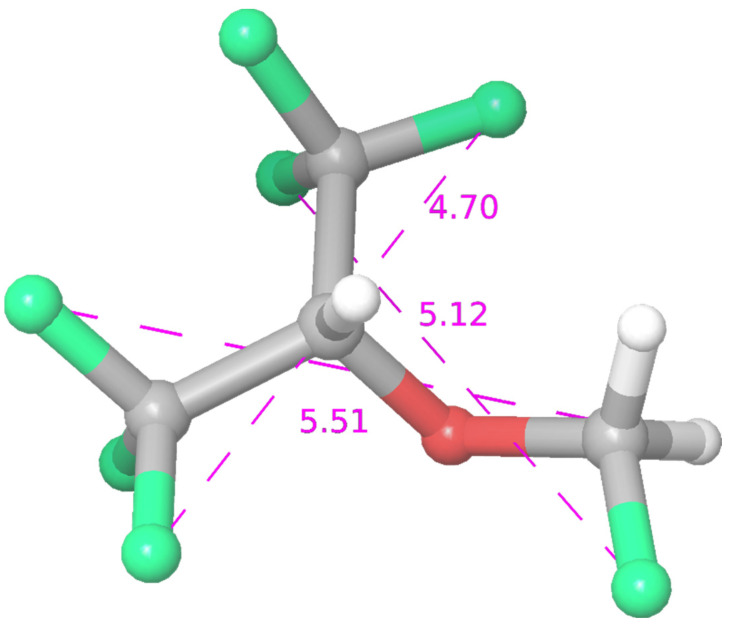
Schematic drawing of sevoflurane.

Sevoflurane (SF) is sweet-smelling non-flammable highly fluorinated methyl isopropyl ether with a boiling point at a temperature of 58.5 °C. Molar mass is 200.055 g/mol, vapor pressure 197 mmHg at 26 °C, and dipole moment 2.33 D [1]. It is one of the most commonly used inhalational anaesthetics, and it has been widely investigated. The recent review on inhaled anaesthetics [2] covered their environmental role, occupational risk, and clinical use. Gaya da Costa and co-authors [2] extensively elaborated on the case of sevoflurane molecule and its contribution to the global warming effect as a volatile anaesthetic (especially in combination with the use with N_2_O), pointed out that its threshold has not yet been established in the workplace as waste anaesthetic gas, considering its side effects in a clinical context (epileptiform electroencephalogram patterns in both adults and paediatric populations; the cardio protective effect in patients with coronary artery disease undergoing vascular surgery, kidney transplantation or lung surgery). The atmospheric lifetimes of the halogenated anaesthetics, halothane, enflurane, sevoflurane, isoflurane, and desflurane to the reaction to the hydroxyl radical (OH) and UV photolysis have been determined from observations of OH reaction kinetics and UV absorption spectra [3]. Halothane, enflurane, and isoflurane showed distinct UV absorption in the range 200–350 nm, and no absorption in the wavelength range 200–350 nm was detected for sevoflurane. Tang et al. [1] analysed the effects of general anaesthetics on their potential targets by large-scale molecular simulation. The structural parameters and partial atomic charges of the anaesthetics, considered to be of great importance, were determined. Geometric optimizations using the Hartree–Fock and the B3LYP (Becke, 3-parameter, Lee–Yang–Parr) density functional theory methods with the large 6–311+G (2d,p) basis set were performed to determine the structures and charge distributions of two halogenated anaesthetics; sevoflurane and halothane. Lesarri and co-authors [4] investigated the conformational landscape of the volatile anaesthetic sevoflurane. The structure of the anaesthetic haloether sevoflurane (CH_2_F–O–CH(CF_3_)_2_) has been resolved using Fourier-transform microwave (FT-MW) spectroscopy in a supersonic-jet expansion. In isolated conditions, sevoflurane adopts a single conformation characterized by a gauche fluoromethoxy group and a near-symmetric orientation of the isopropyl group with respect to the ether plane (cis H–Cipr–O–CF). A schematic drawing of sevoflurane is shown in Figure 1.

Don et al. [5] proposed a vibrational assignment of sevoflurane and studied its interaction with the aromatic model compound benzene, using the vibrational spectroscopy of supersonic jet expansions and of cryo solutions in liquid xenon. Sevoflurane has been investigated from the medical point of view also. Shiraishi and Ikeda [6] studied an uptake and biotransformation of sevoflurane in humans and concluded that, despite its relatively large minimum alveolar concentration (MAC), sevoflurane has a small uptake due to its low solubility, but the degradation rate is shown to be high, resulting in a higher serum fluoride concentration than for other halogenated anaesthetics. Most recently, Dong et al. [7] assessed mice and neurons treated with anaesthetics sevoflurane and desflurane, and applied nanobeam-sensor technology, an ultrasensitive method, to measure tau/p-tau amounts. They showed that the sevoflurane induces tau trafficking from neurons to microglia.

Lange et al. [8] have investigated the lowest-lying electronic states of isoflurane and sevoflurane in the 5.0–10.8 eV energy range by experimental and theoretical methods. Photoabsorption spectra of isoflurane and sevoflurane have been measured with synchrotron radiation over the photon range 5.0–10.8 eV. Low-lying excited singlet valence and Rydberg states are investigated, and the assignments supported by quantum chemical calculations, the latter also helping to identify the triplet states. The measured absolute cross-sections have been used to calculate the photolysis lifetimes of isoflurane and sevoflurane in the Earth’s atmosphere.

Electron collisions with sevoflurane have also been investigated. Very recently, Lozano et al. [9] reported total scattering cross-section from sevoflurane of 1–300 eV electrons. The experimental results, obtained from an implemented magnetic beam apparatus, are compared with theoretical results from the independent atom model, with a screening corrected additivity rule including interference effects and rotational excitation.

As pointed out by Tanaka et al. [10], the absolute electron-impact excitation cross-sections could be obtained by normalization to elastic scattering cross-section (which is the subject of the present study), or in other ways to determine the binary encounter (BE) scaling curve, after measuring the DCSs at high-energy impact (>100 eV), and for small scattering angles. A comprehensive review of experimental techniques and calculation methods for determination of electron cross-sections from biomolecules, biofuels and their fragments, has been recently given by Brunger [11], covering extensive literature in the field.

In this paper, theoretical and experimental results for elastic electron scattering from sevoflurane, in the medium energy range, are shown, complementing the investigations of electron interaction with this molecule. According to our knowledge, these are the first reported results for absolute differential cross-sections in the energy range from 50–300 eV.

Experimentally obtained data include absolute differential cross-sections for elastic electron scattering for the incident electron energy range from 50 to 300 eV (with 50 eV steps) and an angular range from 25° to 125° (with 5° steps), and integral and momentum transfer cross-sections for every measured energy. The experiment was performed on a crossed-beam apparatus. Relative points were put on the absolute scale with the help of the relative flow method, using Ar as a reference gas. Theoretical results are obtained using the IAM-SCAR+I method (independent atom model (IAM) applying the screened additivity rule (SCAR) with interferences terms included (I)). A very good level of agreement has been found between the present experimental and theoretical data, within the experimental uncertainties.

The paper is organized as follows. The experimental setup and measurement procedure are given in Section 2. The theory and calculations of DCSs are explained in Section 3. Obtained results are shown graphically and in the table, and discussion is shown in Section 4. Section 5 is reserved for the conclusion.

## 2. Experiment

**Figure 2:**
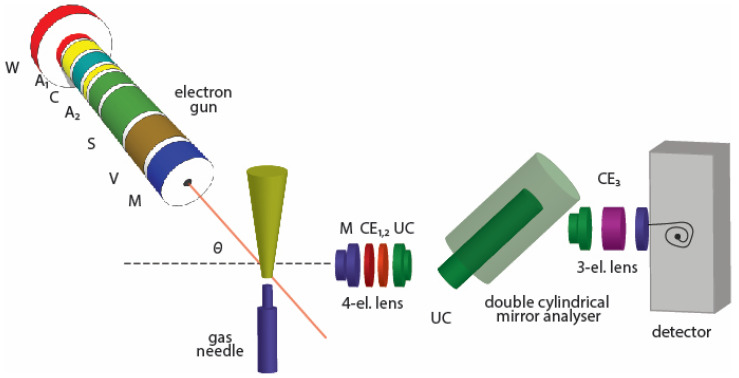
Schematic drawing of the experimental set-up.

A schematic drawing of the experimental set-up is shown in Figure 2. Our beam profile has been determined in a previous set of measurements [12,13,14]. In our experiment, the source of molecular beam is a stainless steel gas needle, with a diameter of d = 0.5 mm and a tube length of l = 40 mm, which gives the ratio Γ = d/l = 80, while the input pressures were such that the free mean path (λ) was larger than the inner diameter of the tube. According to Lucas [15], the proposed conditions for Γ and λ are Γ > 10 and λ ≈ d, so we fulfil all the requirements for the well-collimated beam, and the derived expressions can be used in our experiment. Moreover, author [15] states that the beam properties will be optimized if the ratio of the square of single tube length and its diameter is maximized, which, in our case, is 3200. 

The analysing system consists of a front four-electrode lens, energy analyser, and rear three-electrode lens. The four-electrode lens is used for collecting, directing, and focusing scattered electrons to the energy analyser after slowing them down to the constant analyser pass energy. Electron energy analyser is a double cylindrical mirror analyser (DCMA), which, essentially, represents two cylindrical analysers connected in series. Pass energy is determined by the potential difference between the cylinders. The three-electrode lens is used for focusing analysed electrons into the detector (single channel electron multiplier). Since detecting particles are electrons, detector entrance is grounded, and exit is on a high positive potential.

The anhydrous sevoflurane (C_4_H_3_F_7_O) used in this experiment had a declared purity better than 99%. Before starting the measurements a few freeze-thaw-pump cycles under vacuum have been made. Sevoflurane (SF) is a liquid at room temperature and was introduced into the scattering region from a glass container via a gas line system. SF molecule is characterized by vapor pressure (197 mm Hg at 26 °C) and relatively high dipole moment (2.33 D). Therefore, to provide stable experimental conditions, everything was heated to approximately 50 °C during the gas phase measurements in analogy with previously studied molecular targets [16,17]. In the present work, pipes and the needle were also heated providing a stable driving pressure behind the needle and also for avoiding the absorption effects [18].

In order to reduce magnetic disturbance, the experimental setup is placed in the chamber shielded by the two concentric µ-metal layers. The pre-experimental pressure in the chamber was about 5·× 10^−7^ mbar and the working pressure was an order of magnitude higher.

The experimental procedure for acquiring the absolute differential cross-sections for elastic scattering of electrons from sevoflurane molecule consists of two main stages:

- Stage 1: obtaining relative DCSs by measuring the intensity of electrons elastically scattered from SF, in the function of scattering angle (from 25° to 125° in 5° steps), for a given incident electron energy (50, 100, 150, 200, 250 and 300 eV). Electron intensities are measured at least three times for every incident energy. Background contributions are suppressed by introducing gas into the chamber, away from the interaction volume, via a side leak, and by subtracting measured intensities from the apparent signal for every angle at the given energy;
- Stage 2: Obtaining absolute points (two) for every electron energy, and normalizing relative DCSs on them. Absolute points are obtained using the relative flow method [19,20].

In the present work, DCSs for elastic electron scattering from sevoflurane have been measured at selected incident electron energies, from 50 to 300 eV (in 50 eV steps), and at scattering angles from 20° to 125° (in 5° steps). At given electron energy, the relative cross-section has been derived as a function of scattering angle by measuring the elastic scattering intensity at the maximum of the elastic peak. The background contributions of the elastic electron intensities, which were around 10% at higher energies, were subtracted from the measured electron yields. It should be noted that the background contributions were generally more important at low incident energies and scattering angles (around 15%). During the measurements, potentials on electron gun electrodes and DCMA were adjusted to keep interaction volume constant. Deviations that can occur at small angles are corrected by comparing cross-sections from Ar at given energy with other authors’ data [14,21].

Measured relative DCSs were normalized to the absolute scale according to the absolute points (at 40°, 80°, 90° or 100°), obtained by a Relative flow method (RFM) previously explained in detail [22]. In RFM we compare intensities of elastically scattered electrons on the target molecule (SF) and referent gas (we used Ar), under the same experimental conditions [19,20]. In order to obtain the same experimental conditions, the same beam profiles, for both target molecule and referent gas must be provided. According to Olander and Kruger [23], the same beam profiles can be obtained with two conditions: (1) the mean free paths for both gases must the same and (2) the mean free path is approximately equal to the diameter of stainless steel gas needle. λ ≈ d. In the experiment, the first condition is achieved by adjusting pressures behind the gas needle inversely proportional to the ratio of the squared gas kinetic diameters, and for second condition gas pressures need to be low, which is fulfilled in our experiment [14]. Gas kinetic diameter for Ar is 3.58 Å and for sevoflurane 5.51 Å, so the pressure ratio was P_Ar_: P_SF_ = D^2^_SF_: D^2^_Ar_ ~ 2.4. When the same beam profiles for both, target molecule (*DCS_x_*) and referent gas (*DCS_ref_*) are obtained, according to Nickel at al [19,20], absolute differential cross-sections for target molecule *DCSx* (*E*, *θ*) can be calculated, knowing absolute differential cross-sections for the referent gas *DCS_ref_* (*E*, *θ*), at the same energy and angle, using a formula:

(1) $$DCS_{x}\left(E,\theta \right)=DCS_{ref}\left(E,\theta \right)\frac{N_{x}F_{ref}}{N_{ref}F_{x}}\sqrt{\frac{M_{ref}}{M_{x}}}$$

Quantities that we measure in the experiment are: Flow rate (F) and intensity of elastically scattered electrons (N). *M_ref_* and *M_x_* are molecular masses for referent gas and target molecule, respectively. The relative flow rate has been determined by closing an outlet to the chamber, admitting target gases into a closed constant volume, and then measuring the pressure increase in time. The flow is determined from the experimental curve of pressure versus time fitted by the least-squares method. The whole system was heated to minimize absorption effects [18], which can influence measuring flow rates and determining the flow.

Our experimental spectrometer UGRA is limited in performing angular dependent differential cross-sections measurements (25–125°) and to obtain integral (*ICSs*) and momentum transfer cross-sections (*MTCSs*) we need to extrapolate measured *DCSs* to 0° and 180°. For extrapolation, from 0–25°, and 125–180° we used calculated results obtained by IAM + SCAR + I procedure (see section Theory) and appropriate integration. Our experimental *DCS* data are obtained in a rather limited angular range and that is why the presented integral cross-sections depend strongly upon the extrapolation procedure. The exact formulae used for the integration are in the form:

(2) $$ICS=2\pi \int_{0}^{\pi }DCS\left(\theta \right)\sin\theta d\theta$$

(3) $$MTCS=2\pi \int_{0}^{\pi }DCS\left(\theta \right)\left(1-\cos\theta \right)\sin\theta d\theta$$

The uncertainties of the relative *DCSs* consist of statistical uncertainties and short-term stability uncertainties, caused by the instability of the system. This uncertainty is increased by 20% for small scattering angles, due to the potential alteration of the interaction volume. Dominant uncertainty for absolute *DCSs* are those from reference cross-sections for Ar [14,21], and are taken to be about 20%. The total uncertainties, derived as the square root of the sum of squares of individual independent uncertainties, for absolute *DCSs* are about 30% for small angles and about 22% for the rest of the angular range. The total uncertainties of *ICSs* and *MTCs* arise from the *DCSs* uncertainties mentioned above and uncertainties of the extrapolation of *DCSs* to 0° and 180° and numerical integration and were approximately 30%.

## 3. Theory

To calculate the differential elastic, integral elastic, and inelastic as well as the total cross-sections we have used the IAM-SCAR+I method (independent atom model (IAM) applying the screened additivity rule (SCAR) with interferences terms included (I)). This method has been described in detail in previous publications [9,24,25,26,27,28]. Briefly, the molecular target is described as an aggregate of its individual atoms (i.e., C, H, F, and O in this case). Each atomic target is represented by an ab initio interacting complex optical potential given by:

(4) $$V_{opt}(\vec{r})=V_{R}(\vec{r})+iV_{abs}(\vec{r})$$

In Equation (4), the real part accounts for elastic scattering while the imaginary part represents the inelastic processes which are considered as the ‘absorption part’ following the procedure of Staszewska et al. [29]. The real part is divided into three terms that include:

(5) $$V_{R}(\vec{r})=V_{s}(\vec{r})+V_{ex}(\vec{r})+V_{pol}(\vec{r}),$$

where vs. represents a static term derived from a Hartree-Fock calculation of the atomic charge distribution [30], *V_ex_* an exchange term to account for the indistinguishability of the incident and target electrons [31], and *V_pol_* a long-range polarization term [32].

The molecular cross-sections are obtained from the atomic data by using the screening corrected additivity rule (SCAR) procedure [33], incorporating interference (I) term corrections [25], by summing all the relevant atomic amplitudes, including the phase coefficients. In this approach, we obtain the molecular differential scattering cross-section (DCS), which integrated over all the scattered electron angular range gives the integral scattering cross-section (ICS). Moreover, by taking the sum of the ICS for all open channels (elastic ICS and inelastic ICS) the total cross-section (TCS) could be obtained. Note that we do not include at this stage any contribution from vibrational and rotational excitation processes.

Sevoflurane is a polar molecule with a permanent dipole moment of 2.22 D [1] and therefore rotational excitations, not accounted for by the above procedure, are relevant within the scattering scheme. In order to approximately include differential and integral rotational excitation cross-section in the present study, we used the first-Born approximation, following the procedure described in [27]. In these conditions, these cross-sections can easily be calculated by considering the molecule as a rigid rotor, with the initial rotational excited state distribution in equilibrium at 300 K, and calculating all the transitions ΔJ = ±1 (J being the rotational quantum number) assuming the Born approximation but including the correction for the larger angles given by Dickinson (see ref. [34] and references therein).

## 4. Results

**Figure 3:**
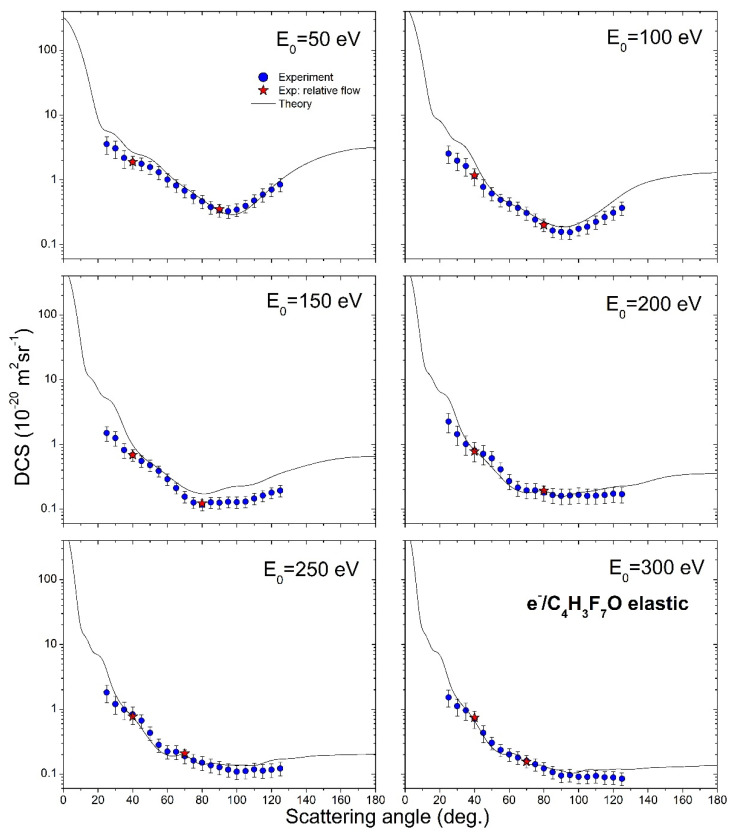
Angularly dependent differential cross-sections for elastic electron scattering from sevoflurane molecule, for six incident electron energies, from 50 eV to 300 eV. The present results include: experiment (blue circles), relative flow absolute data (red stars) and theory (full line).

Experimental results for DCSs, together with experimental ICSs and MTCSs, for elastic electron scattering from sevoflurane, are presented in Table 1, all with corresponding absolute uncertainties. Experimentally measured and theoretically calculated DCSs are shown graphically in Figure 3. The experiment covers six energies of impact electrons, from 50 eV to 300 eV, and the angular range from 25° to 125° (theory covers all angles, from 0° to 180°). Absolute uncertainties are about 22%, except for small angles, where uncertainties are increased to about 30% due to the potential interaction volume alterations.

DCSs have characteristic behavior for molecular targets, noticed previously [12,13]. At 50 eV and 100 eV DCSs exhibit a wide minimum at 100° scattering angle, which disappears at higher incident electron energies. Experiment and theory are generally in good agreement, both in shape and on the absolute scale. There are systematic discrepancies at small angles for all electron energies, probably because of the instability of interaction volume at those angles during the experiment. Also, there are obvious declinations of DCSs for 150 eV electron energies at high scattering angles.

Concerning the normalization procedure of our results, in the relative flow measurements, absolute DCSs for Ar are taken from Ranković et al. [14] for incident electron energies 50–200 eV and 300 eV and from Williams and Willis [22] for electron energy of 250 eV. In both papers, the absolute values were derived by measurements of angular dependences of elastically scattered electrons using electron spectrometers, two 127° cylindrical electrostatic energy analysers in [21], and a double cylindrical mirror analyser (DCMA) in [14]. Authors had different normalization procedures. Ranković et al. [14] used He as a reference gas and Williams and Willis [21] a phaseshift analysis of the relative angular distributions of electrons elastically scattered in the energy region of the resonances ^2^P_3/2.1/2_ of Ar. Both absolute differential cross-sections agree within mutual uncertainties as discussed in [10]. Since our normalization is based upon RFM, we prefer to use values from the most recent paper [14] because they were obtained at the same electron spectrometer UGRA and only for the energy of 250 eV, which is not available from our apparatus [14], we used those from Williams and Willis [21].

The shapes of the experimental and theoretical cross-section plots are in excellent agreement, but measured points are 25% lower than the calculated ones. Because our experiment is angularly limited, ICS depends on the used extrapolation method, which is consisted of the normalization of our theoretical DCSs to our measured absolute ones. We have normalized calculated DCS to the measured absolute data to best match in shape and then used these values for integration. These values are presented in Table 1 with an uncertainty of 30% that arises from different plausible extrapolations. The absolute uncertainties of ICS and MTCS values are obtained from the difference of the corridor that is represented by maximal, DCS + Δ/2, and minimal DCS − Δ/2 values.

**Figure 4:**
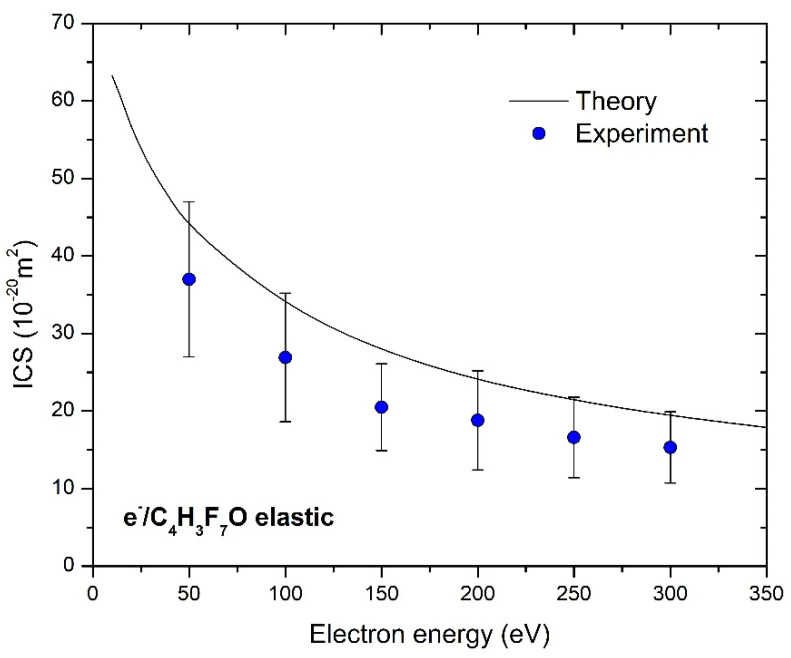
Integral cross-section for elastic electron collision with sevoflurane molecule in the energy range from 10 eV to 350 eV. Experiment (blue circles) and theory (solid black line) are presented.

Measured and calculated absolute differential and integral cross-sections are graphically presented in Figure 3 and Figure 4, and to the best of the authors knowledge, there are no other published results, neither experimental nor theoretical, for DCS or ICSs for elastic electron scattering from sevoflurane in this energy and angular range.

## 5. Conclusions

We performed the experimental and theoretical investigation of elastic electron collisions on sevoflurane molecule at intermediate incident electron energies in order to provide insight into electron/sevoflurane interaction. Two independent sets of measurements, relative differential cross-sections at fixed energy in the function of scattering angle and absolute differential cross-sections obtained by relative flow method were performed. The last one provides us with two absolute points for every incident energy, which were used for the normalization to the absolute scale. These two independent sets of measurements showed good agreement which gives reliability to our experimental procedure. We have shown good agreement between the present experiment and theory (IAM-SCAR+I method) on the absolute scale and also in the shape. Using Ar we stress the problem of the choice of the reference gas in the relative flow measurements, since conditions should be provided for the widths (shapes) of the target beam and the reference gas beam to be approximately the same, which is easier to achieve with Ar in relation to He or Ne, since they are much lighter. The importance of this investigation is that according to our knowledge these are the first reported results for absolute differential and integral cross-sections for elastic electron scattering on sevoflurane in the energy range from (50–300) eV.

## Figures and Tables

**Table 1:** Experimental results for absolute differential cross-sections (DCSs), integral cross-sections (ICSs) and momentum transfer cross-sections (MTCSs) for elastic electron scattering from sevoflurane. In parentheses are given absolute uncertainties of the last two digits.

θ (°)	DCS (10^−20^ m^2^ sr^−1^)					
	50 (eV)	100 (eV)	150 (eV)	200 (eV)	250 (eV)	300 (eV)
25	3.6(1.1)	2.55(76)	1.50(39)	2.25(74)	1.82(56)	1.53(45)
30	3.07(93)	1.98(59)	1.25(32)	1.44(48)	1.20(37)	1.12(33)
35	2.17(66)	1.64(49)	0.82(21)	1.01(33)	0.99(30)	0.96(28)
40	1.89(43)	1.14(34)	0.69(14)	0.80(26)	0.84(26)	0.72(21)
45	1.77(40)	0.78(23)	0.55(11)	0.71(24)	0.67(16)	0.43(13)
50	1.57(36)	0.61(14)	0.476(94)	0.61(16)	0.43(10)	0.304(66)
55	1.31(30)	0.49(11)	0.386(77)	0.41(11)	0.282(67)	0.235(51)
60	1.01(23)	0.428(98)	0.289(57)	0.270(71)	0.223(53)	0.202(44)
65	0.82(18)	0.369(84)	0.211(42)	0.214(57)	0.220(52)	0.180(39)
70	0.68(15)	0.307(70)	0.155(31)	0.194(51)	0.189(45)	0.160(35)
75	0.55(13)	0.243(56)	0.126(25)	0.195(51)	0.162(38)	0.141(31)
80	0.47(11)	0.203(47)	0.117(23)	0.179(47)	0.150(36)	0.122(27)
85	0.380(86)	0.165(38)	0.127(25)	0.165(44)	0.139(33)	0.107(24)
90	0.339(77)	0.157(36)	0.125(25)	0.160(43)	0.127(30)	0.095(21)
95	0.327(74)	0.154(35)	0.129(26)	0.162(44)	0.117(28)	0.096(21)
100	0.347(79)	0.175(40)	0.128(25)	0.167(44)	0.109(26)	0.091(20)
105	0.396(90)	0.188(43)	0.130(26)	0.160(42)	0.111(27)	0.090(21)
110	0.48(11)	0.225(51)	0.146(29)	0.160(42)	0.118(28)	0.093(20)
115	0.59(13)	0.265(61)	0.161(32)	0.165(44)	0.113(27)	0.089(20)
120	0.71(16)	0.311(71)	0.179(35)	0.173(46)	0.116(28)	0.088(20)
125	0.85(19)	0.367(84)	0.193(38)	0.170(45)	0.122(29)	0.085(19)
ICS’s	37(10)	26.9(8.3)	20.5(5.6)	18.8(6.4)	16.6(5.2)	15.3(4.6)
MTCS’s	13.5(4.3)	5.8(1.5)	2.94(68)	2.90(85)	2.14(58)	1.66(43)

## Data Availability

The data presented in this study is contained within the article. All data are possible to retrieve from the Belgrade Electron Atom/Molecule DataBase (BEAMDB) at http://servo.aob.rs/emol after the article has been published.
